# Assessment of bladder filling during prostate cancer radiation therapy with ultrasound and cone-beam CT

**DOI:** 10.3389/fonc.2023.1200270

**Published:** 2023-07-27

**Authors:** Kiran Chauhan, Daniel K. Ebner, Katherine Tzou, Karen Ryan, Jackson May, Tasmeem Kaleem, Daniel Miller, William Stross, Timothy Dean Malouff, Robin Landy, Gerald Strong, Steve Herchko, Chris Serago, Daniel Michael Trifiletti, Robert Clell Miller, Steven Buskirk, Mark R. Waddle

**Affiliations:** ^1^ Department of Radiation Oncology, Mayo Clinic, Rochester, MN, United States; ^2^ Department of Radiation Oncology, School of Medicine, University of Colorado Anschutz Medical Campus, Aurora, CO, United States; ^3^ Department of Radiation Oncology, Mayo Clinic, Jacksonville, FL, United States; ^4^ College of Medicine, Florida State University, Tallahassee, FL, United States; ^5^ Department of Radiation Oncology, Trihealth System, Cincinnati, OH, United States; ^6^ Department of Radiation Oncology, Gamma West Cancer Center, Idaho Falls, ID, United States; ^7^ Department of Radiation Oncology, United States Department of Veterans Affairs, Minneapolis, MN, United States; ^8^ Department of Radiation Oncology, College of Medicine, University of Oklahoma, Oklahoma City, OK, United States; ^9^ Department of Therapeutic Radiology, School of Medicine, Yale University, New Haven, CT, United States; ^10^ Department of Radiation Oncology, University of Tennessee Medical Center, Knoxville, TN, United States

**Keywords:** bladder filling, IMRT, radiotherapy, CBCT, workflow

## Abstract

Prostate cancer patients undergoing external beam radiation therapy (EBRT) benefit from a full bladder to decrease bowel and bladder toxicity. Ultrasound may offer a proxy metric for evaluation, sparing CBCT dosing. Patients were prospectively enrolled pre-simulation from January 2017 to February 2018. Bladder volume was evaluated prior to RT using US daily and CBCT for three daily treatments and then weekly unless otherwise indicated. 29 patients completed median 40 days of RT, resulting in 478 CBCT and 1,099 US bladder volumes. 21 patients were treated to intact glands and 8 to the post-prostatectomy bed. Median patient age was 70 years. Bladder volume on CBCT and US positively correlated (r = 0.85), with average bladder volume for all patients of 162 mL versus 149 mL, respectively. Bladder volume during treatment was consistently lower than the volume at CT simulation (153 mL vs 194 mL, p<0.01) and progressively declined during treatment. Patients older than 70 years presented with lower average bladder volumes than those < 70 years (122 mL vs 208 mL, respectively, p<0.01). Patients with the highest agreement between CBCT and US (<10% variability) had higher average bladder volumes (192 mL vs 120 mL, p=0.01). US was found to be an accurate measure of bladder volume and may be used to monitor daily bladder volumes in patients being treated with radiation for prostate cancer.

## Introduction

In the modern era, radiation oncologists use highly conformal, high dose radiation for the treatment of prostate cancer. To achieve higher treatment doses with intensity-modulated radiation therapy (1), careful attention is paid to the surrounding normal structures and efforts are made to decrease dose to the surrounding tissue. One such effort is daily consistent filling of the bladder to distend a portion of the bladder outside of the treatment field, to fill the lower pelvis, reduce small bowel entering the radiation field, and to improve reproducibility of radiation dose to the bladder and rectum. Patients with prostate cancer face unique challenges regarding bladder filling due to symptoms related to their disease such as urinary frequency, urgency, incontinence, or urinary retention. Further, many patients are treated in the post-prostatectomy setting with decreased urinary control. Bladder filling after CT simulation is patient-dependent often with limited feedback available to patients as to the success and reproducibility of their efforts.

Several studies have previously investigated the reproducibility of bladder filling on radiation treatments for prostate cancer and have found bladder filling to be suboptimal due to variation in bladder volume during treatment (2, 3). Variation in bladder size has been shown to be related to significant motion of the organs at risk, such as the bladder and rectum, as well as risk of geographic misses in post-prostatectomy radiation. These studies have all used cone beam CT (CBCT) to evaluate bladder volume, which is often performed at the time of treatment for treatment position verification. However, CBCT is not always used for daily objective assessment of bladder volume in clinical practice and can be technically limited due to a small field of view. In many cases, the patient’s sense of bladder fullness is relied on, and suboptimal bladder filling is not identified until a patient is on the table in the treatment position during the alignment CBCT. This requires the patient to leave the treatment room, drink fluids and/or wait for bladder filling, then return for another CBCT. This delay can result in significant reductions in efficiency of a practice, delays for other patients, and reduce the throughput of a treatment machine.

One alternative to CBCT is assessment of bladder motion via rapid bladder ultrasound. This simple test is non-invasive and can be performed quickly on the treatment table prior to CBCT, potentially allowing faster feedback for patients and improved bladder filling consistency. We hypothesize that this would allow for improved throughput and limit patient delays. Bladder volumes assessed with bladder scanning have shown to correlate well with bladder volumes on CT imaging. The primary aim of this study was to conduct a pilot study to assess the variation in bladder filling volumes using bladder ultrasound in a cohort of patients with prostate cancer and to compare these measurements to those on CBCT. In patients with prostate cancer, we hypothesized that the rapidity of bladder ultrasound may allow for acceleration of treatment workflow, quickly identifying those patients with poor or inaccurate filling prior to treatment positioning.

## Materials and methods

This was a prospective single arm study of adult patients being treated with definitive or salvage radiation therapy for prostate cancer at Mayo Clinic. Patients receiving both conventional and hypo-fractionated radiation were included. Patients with a large body habitus that limited ultrasound readings were excluded. IRB approval was obtained prior to initiating this study and all patients enrolled gave full written consent for participation.

### Bladder filling instructions

Bladder filling was conducted per standard institutional protocol and patients were blinded to all bladder ultrasound readings to avoid the introduction of bias. At the time of CT simulation, patients had a catheter placed into the bladder and 200 mL of saline-diluted contrast instilled, with a soft penile clamp placed to ensure no leakage. For daily treatment, patients were instructed to present with a comfortably full bladder and were specifically told to drink 16 ounces of water 30-60 minutes prior to treatment. Patients were then told to adjust the volume and timing of water intake based on their sense of bladder fullness at the time of treatment. The patients were routinely asked about bladder filling during on-treatment visits in line with prior standard of care, and bladder ultrasound values were blinded to providers.

### Bladder ultrasound

Bladder ultrasound measurements were collected by radiation therapists on the treatment table, prior to CBCT alignment. Radiation therapists were trained in the use of this device by an experienced physician assistant from the Department of Urology. Care was taken to record bladder volume on the lower abdomen with the ultrasound probe pointing inferior and posteriorly, approximately 5 cm below the umbilicus. When possible, the same radiation therapist performed bladder ultrasound measurements throughout the course of treatment. Three consecutive bladder ultrasound readings were collected at the time of CT simulation and daily at each treatment. The three bladder volumes were averaged to give the measurement for each day. Radiation therapists were instructed not to share this information with the patient.

### Cone beam CT

**Figure 1:**
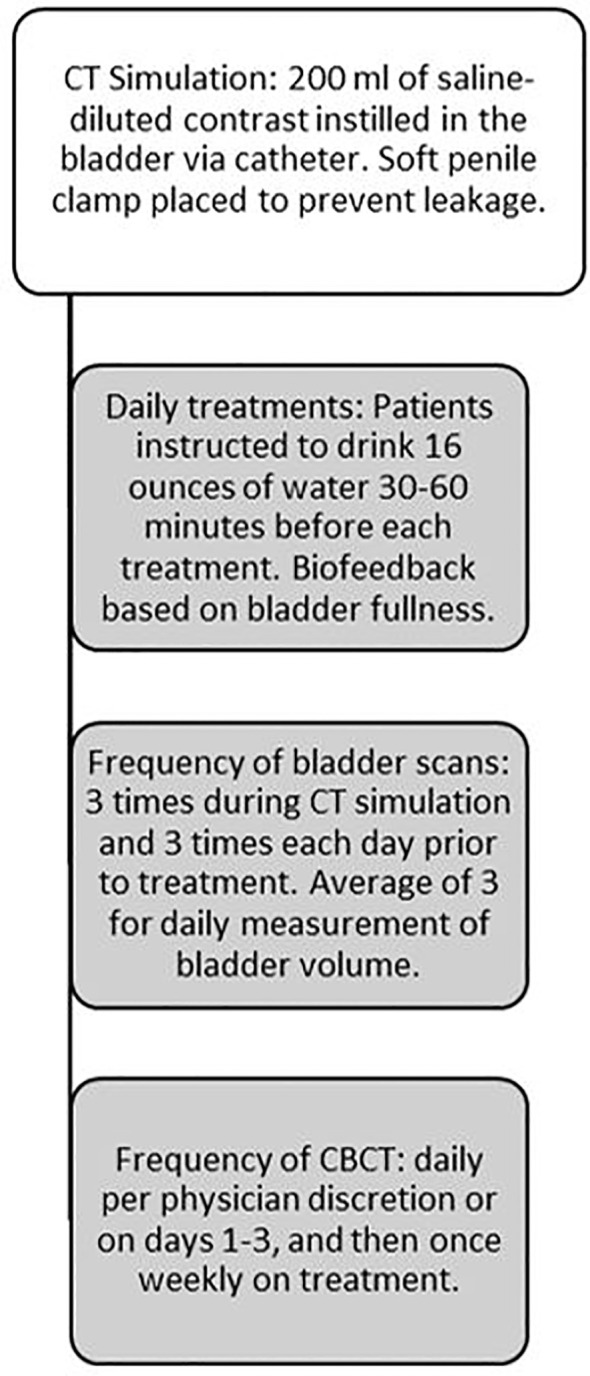
Bladder Filling Protocol Outline.

CBCT was performed during treatment to serve as the gold standard for defining bladder volumes. Patients received daily CBCT at the discretion of the treating physician; otherwise, CBCT was performed on days 1-3, and then once weekly while the patient was under treatment. Bladder volumes were contoured in a blinded manner on the CBCT image sets by a single radiation oncologist using the interior bladder wall as the outside of the bladder volume and the volumes were recorded. An outline of the bladder filling protocol can be seen in **Figure 1**.

### Statistical analysis

Descriptive statistics were used to describe bladder filling volumes and variation over time. Student’s t-test was used to compare bladder volume between patient groups. The relationship between CBCT and ultrasound measurements were evaluated using Pearson correlation. One way ANOVA was used to compare bladder volumes between patients grouped into normal weight (BMI of 18.5 to 24.9), overweight (BMI of 25 to 29.9), and obese (BMI > 30) categories. Excel statistical functions (Microsoft) were used for statistical analysis. A p-value < 0.05 was considered significant.

## Results

**Figure 2:**
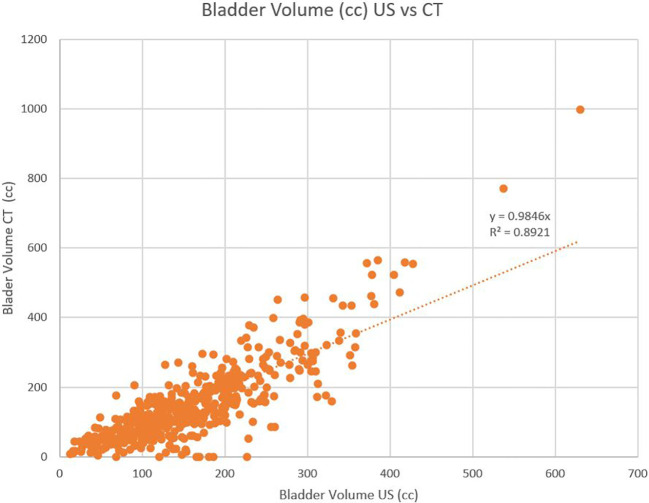
Bladder volume correlation between CT and US.

**Figure 3:**
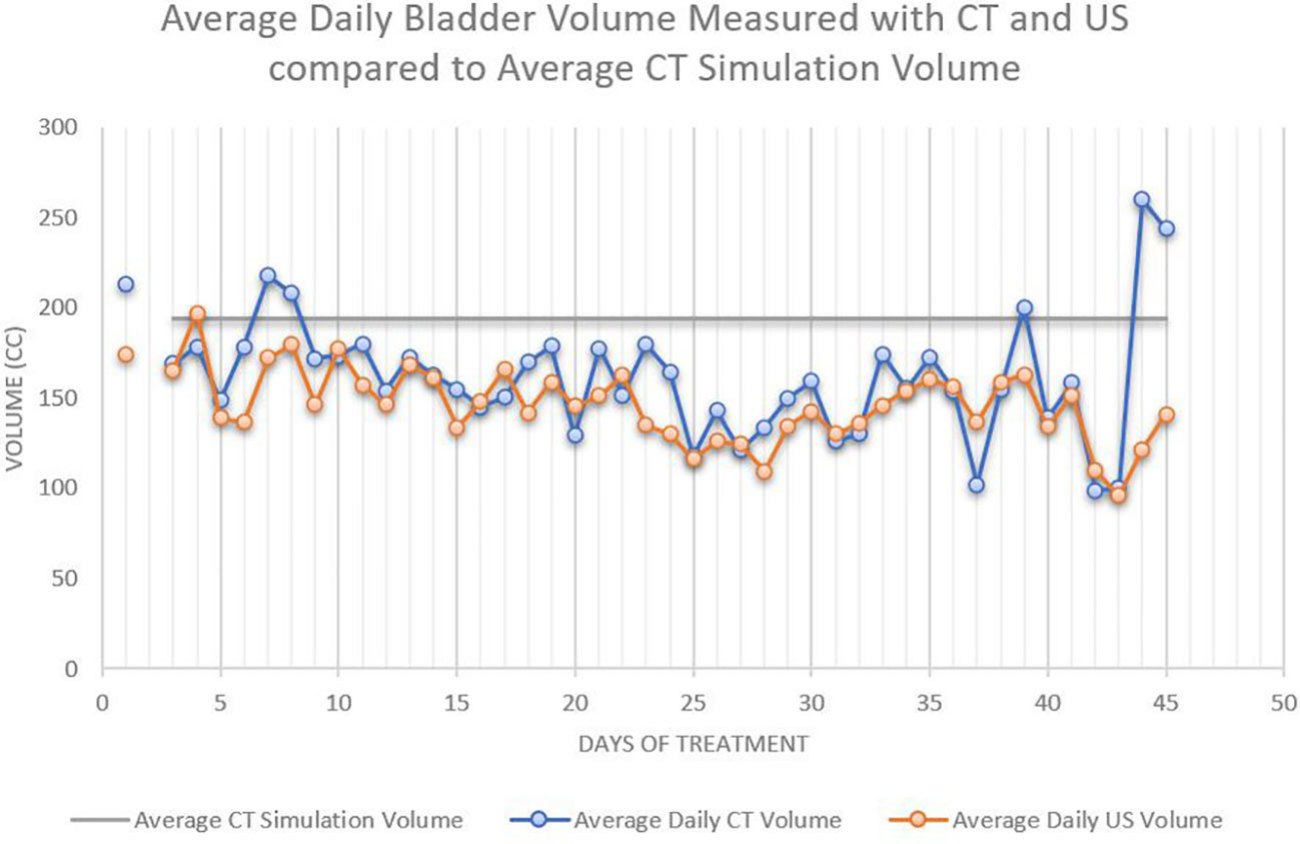
The average daily bladder volumes were lower than the average bladder volume during CT simulation, 194 cc. The average bladder volumes decreased with increasing number of treatments.

A total of 29 patients completed a median of 40 days of RT from January 2017 to February 2018, resulting in 478 CBCT and 1,099 US bladder volumes. Twenty-one patients were treated to intact glands and eight to the post-prostatectomy bed. Patients ranged from 57 to 87 years old, with the median age of 70 years. Patient demographics are included in **Table 1**. There was a positive correlation between bladder volume on CBCT and US (R^2 = ^0.892 and r=0.85) with average bladder volume for all patients of 162 mL versus 149 mL, respectively (**Figure 2**). Bladder volume during treatment was consistently lower than the volume at CT simulation (153 mL vs 194 mL, p<0.01) and progressively declined during treatment, with an average volume during treatments 1-10 of 164 mL vs treatments 11-44 of 141 mL (p<0.01) (**Figure 3**). Patients older than 70 years presented with lower average bladder volumes than those < 70 years (122 mL vs 208 mL, respectively, p<0.01). Average bladder volume did not differ by baseline AUA score (p=0.43) or by pre- or post-op treatment (p=0.11). Additionally, there was no significant difference in average bladder volumes based on BMI (p=0.53). Patients with the highest agreement between CBCT and US (<10% variability) had higher average bladder volumes (192 mL vs 120 mL, p=0.01).

**Table 1:** Patient demographics.

	n	%
Age		
Median Range	70 [57-87]	
BMI		
Normal 18.5 to 24.9 Overweight-25-29.9 Obese <30	4 16 9	14% 55% 31%
T stage		
T1 T2 T3 n/a	9 12 7 1	31% 41% 24% 3%
Gleason Score		
6 3+4=7 4+3=7 8+ n/a	2 6 4 14 1	7% 21% 14% 55% 3%
Grade		
Low Risk Intermediate Risk High Risk n/a	1 5 18 3	3% 17% 62% 17%
Treatment to:		
Intact prostate Post-prostatectomy Bed	21	72% 28%
Number of Treatments		
Median Range	40.5 [20-44]	
Baseline AUA*		
symptom scores		
Mild (0-7) Moderate (8-19) Severe (20-35) n/a	17 8 3 1	59% 28% 10% 3%

*American Urological Association symptom scores to assess for baseline urinary dysfunction in men.

## Discussion

This study showed that bladder US is a reasonable estimate for bladder volume on CBCT, demonstrating positive correlation (r=0.85), and identified variation in bladder filling in older patients, but no variation based on BMI or AUA symptom score. Therefore, bladder ultrasound may be a fast and reliable method to estimate bladder volume prior to treatment to reduce unnecessary CBCTs, improve efficiency of treatment delivery, and to improve dose to the target and organs at risk. Further, bladder ultrasound may be most useful in older patients who demonstrated the most variation over the course of treatment.

These findings are important since variations in bladder volumes have been shown to change dose distribution to targets and organs at risk. Rosewall and colleagues evaluated this in 35 prostate cancer patients undergoing IMRT. The patients had CBCTs every third treatment, including biomechanical modeling to reconstruct bladder dose for the treatment position of the bladder (4). They showed that the reconstructed dose to the bladder was significantly higher when accounting for bladder variation. This was particularly true for bladder volumes >350cm^3^ at the time of CT simulation, with reconstructed volumes at 30 Gy, 65 Gy and 78 Gy demonstrating an absolute difference of 43 cm^3^, 14 cm^3^ and 3 cm^3^, respectively, compared to planning volumes. The authors concluded that the actual dose delivered was larger than planned and that some patients may have higher risk of toxicity than previously thought.

Another study of salvage radiation to the prostate bed showed that variations in bladder filling resulted in changes to the prostate bed coverage, particularly in the superior prostate bed. This study of 40 patients with weekly CBCT showed that surgical clips moved significantly and were associated with changes in bladder volume (5). They showed that these changes resulted in prostate bed “geographic misses” in 62% of patients when the bladder was 2cm higher superiorly and 25-26% when the bladder was 1+ cm lower inferiorly. Nakamura et al. used megavoltage CT during the first, 10th, 20th, and 30th fraction of external beam radiation for prostate cancer to assess the bladder volume. This identified that bladder volume progressively decreased to 62% of that at the time of CT simulation with significant intra-patient variation of 38% (6).

A study of 23 patients with prostate cancer undergoing definitive IMRT to the prostate used daily CBCT to assess bladder size in left-right (LR), anterior-posterior (AP), and superior-inferior (SI) directions in patient with endorectal balloon. They found significant changes in the SI and AP dimensions but found that these changes did not impact the target volume position (7). This study suggests that more information is likely obtained from CBCT than is needed to assess for bladder size, namely regarding spacial organization of surrounding structures. Bladder ultrasounds provide comparable data, without increased radiation exposure from CT, and may be a valid replacement should verification of tissue spacial organization not be required.

Specific to ultrasound, Yoon et al. evaluated the use of bladder ultrasound in 20 patients treated with radiation for rectal cancer to assess bladder volume at the time of treatment and used protocol-based bladder filling education, training, and biofeedback using bladder scan three times weekly during treatment. This study showed that the bladder scan volume correlated well with CT simulation scan (R=0.87) and that patients with education and feedback had a statistically significant decrease in the interquartile range of bladder volume during treatment when compared to a previous cohort without the intervention (8). Cramp et al. implemented a bladder scan protocol for patients undergoing prostate radiation therapy as well, including 34 patients divided between bladder scan (n=17) and non-bladder scan (n=17). Correlation was r=0.797 between CT volume and bladder scan volume. The authors found that the bladder scan group experienced comparatively fewer filling failures requiring patients to be removed from the patient bed when assessing for appropriate bladder volume needed for treatment (9).

In our study, there was a progressive decrease in bladder volume from the time of CT simulation (194 ml) to subsequent treatments (153 ml). Several factors could potentially account for this observation. First, during CT simulation, appropriate bladder filling was attained by instilling 200 ml of saline contrast solution via catheter and placing a penile clamp. Subsequent bladder filling was more subjective, as patients were instructed to drink 16 fluid ounces of water 30 to 60 minutes prior to treatment and adjust their intake for future treatments based on their sensation of bladder fullness. Of note, one limitation of this study relates to the specialized training radiation therapists received. The expertise of the individual measuring bladder volume requires consideration on future implementation of this practice to clinic workflow.

## Conclusions

Alterations in bladder volume can affect radiation dose to target structures and organs at risk for prostate cancer patients undergoing radiation therapy. Bladder ultrasound may be a useful tool to supplement the patient’s subjective report of bladder fullness with a quick, reliable, safe, and objective measurement of bladder volume prior to treatment. In the studied population, older patients and patients with longer treatment courses may benefit most from bladder ultrasound for bladder volume assessment. This study suggests that natural bladder filling at CT simulation, rather than instillation of contrasted saline, may provide the most reproducible bladder volume during treatment.

## Data availability statement

The raw data supporting the conclusions of this article will be made available by the authors, without undue reservation.

## Ethics statement

The studies involving human participants were reviewed and approved by Mayo Clinic Institutional Board Review. The patients/participants provided their written informed consent to participate in this study.

## Author contributions

MW contributed to the conception and design of this study. MW, DE, and KC wrote the first draft of the manuscript. KC generated the figures and tables for the manuscript. KT, KR, JM, TK, DM, WS, TM, RL, GS, SH, CS, DT, RM, and SB contributed to data collection, analysis, and supported other aspects of this prospective study. All authors contributed to the article and approved the submitted version.
